# VISH-DARIJA-TTS: A synthetic moroccan dialect text–audio dataset for vishing and voice-based social engineering detection

**DOI:** 10.1016/j.dib.2026.113087

**Published:** 2026-07-17

**Authors:** Yasser Hmimou, Nada Jabel, Soufiane Ameur, Mohamed Tabaa

**Affiliations:** LPRI Laboratory, Moroccan School of Engineering Sciences (EMSI), Casablanca, Morocco

**Keywords:** Phone fraud, Low-resource Arabic varieties, Speech synthesis, Noisy speech, Multimodal security, Scam conversations, Controlled SNR, Cybersecurity corpus

## Abstract

VISH-DARIJA-TTS v1.0 is a publicly available synthetic multimodal dataset for vishing research in the Moroccan dialect (Darija), a low-resource Arabic variety where publicly documented labelled voice-fraud resources remain highly limited. The dataset was generated through a controlled pipeline comprising scenario authoring, dual-script transcription in Latin-script and Arabic-script Darija, text-to-speech synthesis with Google GenAI Gemini 3.1 Flash TTS, audio normalization, metadata enrichment, deterministic noise augmentation, and release-level validation. It contains 3400 balanced multi-turn scenarios — 1700 normal and 1700 scam/vishing dialogues — with 17,351 dialogue turns. The audio layer includes 3400 clean normalized mono WAV files at 24 kHz and PCM 16-bit, together with 10,200 noisy variants generated at signal-to-noise ratios of 20 dB, 10 dB, and 5 dB, for a total of 13,600 audio files representing approximately 22.61 h of clean audio and approximately 90.45 h when noisy variants are included. The repository includes clean and sanitized text layers, scenario-level and turn-level metadata, heuristic social-engineering labels, emotion and attack taxonomies, audio manifests, train/validation/test splits, SHA-256 checksums, documentation files, and reproducibility scripts. A public text layer replaces sensitive spans with structured placeholders to prevent redistribution of operational social-engineering templates. The dataset contains no real phone calls, real victims, or human-recorded speech. It can be reused for text-based, audio-based, and multimodal vishing detection research, Moroccan Darija speech processing, and controlled noise-robustness benchmarking.

Specifications Table

**Table:** 

Subject	Computer Sciences
Specific subject area	Cybersecurity, speech processing, and low-resource NLP for voice-fraud research
Type of data	Text, audio, metadata, taxonomies, validation reports, scripts, and documentation. Formats: JSON, WAV, CSV, TXT, MD, CFF, PDF, and Python. Processing levels: raw synthetic text, sanitized text, normalized audio, noisy augmented audio, and processed metadata.
Data collection	Data were synthetically generated from balanced Moroccan Darija normal and scam/vishing multi-turn scenarios. Dialogues were represented in Latin and Arabic scripts, converted to speech with Google GenAI Gemini 3.1 Flash TTS configured with the Moroccan Arabic locale (ar-MA), normalized to mono 24 kHz PCM 16-bit WAV, and augmented with 10 Moodist-derived environmental noises at 20, 10, and 5 dB SNR. JSON schema, audio links, splits, and checksums were validated with Python scripts.
Data source location	Multidisciplinary Laboratory of Research and Innovation (LPRI), Moroccan School of Engineering Sciences (EMSI), Casablanca 20,250, Morocco.
Data accessibility	Repository name: Zenodo Data identification number: 10.5281/zenodo.20039126 Direct URL to data: https://zenodo.org/records/20039126 Instructions for accessing these data: The dataset is publicly available through the Zenodo record. Users can access the record through the DOI link, download the release archive, and use the included README.md, dataset_card.md, schema.json, metadata tables, checksums, and scripts to inspect, validate, and reuse the data.
Related research article	None.

## 1 Value of the Data

• The dataset provides a specialized publicly documented Moroccan dialect (Darija) text-audio resource dedicated to vishing and voice-based social engineering research. It combines balanced multi-turn dialogues, dual-script transcription, synthetic speech, noisy audio variants, and structured social-engineering metadata in a single reproducible release.
• The data can support text-based, audio-based, and multimodal research on phone-fraud detection, scam conversation modelling, social-engineering pattern analysis, and cybersecurity-oriented NLP for a low-resource Arabic dialectal variety where publicly documented labelled voice-fraud resources remain highly limited.
• The three-level noise augmentation at 20 dB, 10 dB, and 5 dB signal-to-noise ratio, with fixed seed and deterministic noise assignment, enables controlled and reproducible evaluation of model robustness under acoustic degradation conditions without requiring access to real or proprietary call-centre recordings.
• Scenario-level and turn-level metadata, train/validation/test splits with leakage control, SHA-256 checksums, and automated validation scripts enable independent verification, transparent benchmarking, and straightforward extension to future dataset versions or downstream tasks [[15], [16]].
• The dataset contains no real phone calls, real victims, or human-recorded speech. Its fully synthetic design and public sanitized text layer make it suitable for defensive research, teaching, and reproducible experimentation while preventing redistribution of operational social-engineering templates.

## 2 Background

The Moroccan dialect (Darija) is a low-resource Arabic variety for which NLP resources have addressed written language modelling, lexical resources, translation, and general dialectal processing [[1], [2], [3]].Recent Arabic speech datasets have improved coverage for multidialectal speech recognition, spoken language understanding, and dialectal text-to-speech, but they are not designed around voice-fraud or vishing scenarios in Moroccan Darija [[4], [5], [6], [7]]. In parallel, voice phishing and telecom fraud have received increasing attention in recent cybersecurity research, including text-based, audio-based, and multimodal detection settings [[8], [9], [10], [11]]. However, to the authors’ knowledge, no publicly available resource combines Moroccan Darija dialogue text, synthetic speech, scam/normal labels, social-engineering metadata, and controlled noisy audio in a single release.

VISH-DARIJA-TTS v1.0 was constructed to provide a reusable dataset for defensive research on vishing and voice-based social engineering in Moroccan Darija. The dataset was designed synthetically to avoid collecting real scam calls, victim recordings, or human-recorded speech, while still providing paired text, audio, metadata, splits, validation reports, and reproducibility scripts for controlled experimentation.

## 3 Data Description

**Fig. 1:**
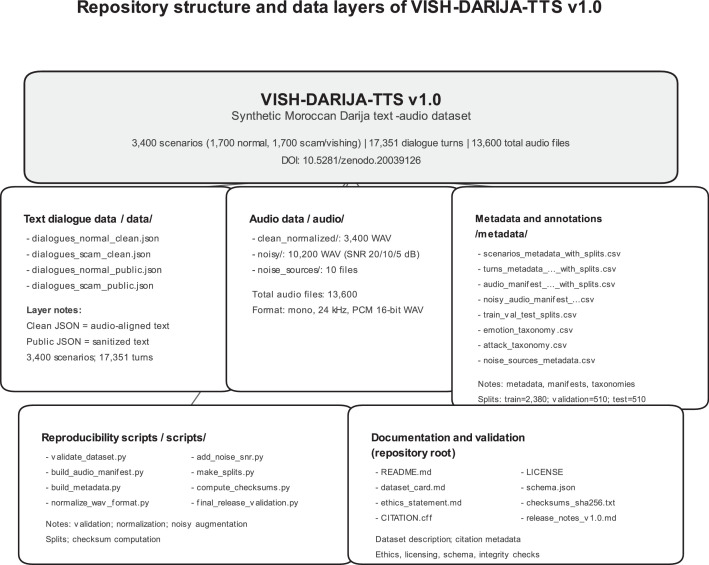
Repository structure and data layers of the VISH-DARIJA-TTS v1.0 dataset. The repository is organized into text dialogue files, clean and noisy audio folders, metadata tables, reproducibility scripts, and documentation/validation files. The clean JSON files provide the text layer aligned with the normalized TTS audio, whereas the public JSON files provide a sanitized text layer for reduced-risk inspection and sharing.

The VISH-DARIJA-TTS v1.0 dataset is publicly available on Zenodo [12]. The repository is organized into text dialogue data, audio data, metadata tables, reproducibility scripts, and documentation/validation files (Fig. 1). The release contains synthetic Moroccan Darija normal and scam/vishing dialogue scenarios, paired clean and noisy audio layers, metadata tables, taxonomies, validation reports, checksums, and reproducibility scripts.

Table 1 summarizes the main dataset composition and audio characteristics.

**Table 1:** Summary of the VISH-DARIJA-TTS v1.0 dataset composition and audio characteristics.

Field	Value
Total scenarios	3400
Normal scenarios	1700
Scam/vishing scenarios	1700
Total dialogue turns	17,351
Clean WAV files	3400
Noisy WAV files	10,200
Total audio files	13,600
Clean audio duration	∼22.61 h
Total audio duration, clean + noisy	∼90.45 h
Audio format	WAV, mono, 24 kHz, PCM 16-bit
Clean duration range	6.88 s – 77.48 s
Clean duration mean	23.94 s
Clean duration median	19.56 s
SNR levels	20 dB, 10 dB, 5 dB
Noise sources	10 Moodist-derived environmental files
Train / validation / test split	2380 / 510 / 510

Table 2 reports the scenario-level distribution of attack_type labels among the 1700 scam/vishing scenarios. Normal scenarios are assigned the attack_type value none and are excluded from the scam-percentage denominator.

**Table 2:** Distribution of attack types across scam/vishing scenarios.

Attack type	Number of scam/vishing scenarios	Percentage among scam/vishing scenarios
fake_bank_support	886	52.12%
other	224	13.18%
family_impersonation	195	11.47%
fake_delivery	84	4.94%
fake_technical_support	63	3.71%
fake_job_offer	55	3.24%
physical_trap	33	1.94%
fake_prize	32	1.88%
marketplace_fraud	27	1.59%
fake_police_or_legal	25	1.47%
fake_donation_or_inheritance	16	0.94%
fake_medical_or_health	16	0.94%
financial_investment_scam	14	0.82%
emotional_manipulation	10	0.59%
travel_or_religious_trip_scam	8	0.47%
extortion_or_blackmail	6	0.35%
fake_admin_or_government	4	0.24%
fake_social_media_or_account	2	0.12%

The distribution was computed from the scenario-level metadata and is intended to document dataset composition rather than to represent the prevalence of real-world scam categories.

Table 3 summarizes the predefined Gemini TTS voices configured with the Moroccan Arabic locale (ar-MA) and their reconstructed distribution across the clean and noisy audio layers. Because no per-scenario voice manifest was stored in the original release, the voice-usage counts were reconstructed by replaying the documented deterministic voice-assignment rule against the clean JSON files and audio manifest. The counts therefore indicate voice-file appearances and are not additive across voices, because a multi-speaker dialogue file may contain more than one predefined voice.

**Table 3:** Distribution of predefined Gemini TTS voice usage configured with Moroccan Arabic locale (ar-MA).

Voice identifier	Locale	Voice configuration/type	Intended role use	Clean files containing voice	Noisy variants containing voice	Total voice-file appearances
Fenrir	ar-MA	Predefined Gemini voice, male pool	Male speakers and scam Speaker 1 when gender is unknown	1335	4005	5340
Puck	ar-MA	Predefined Gemini voice, male pool	Male speakers and scam Speaker 1 when gender is unknown	1296	3888	5184
Orus	ar-MA	Predefined Gemini voice, male pool	Male speakers and scam Speaker 1 when gender is unknown	1281	3843	5124
Charon	ar-MA	Predefined Gemini voice, male pool	Male speakers and scam Speaker 1 when gender is unknown	1326	3978	5304
Kore	ar-MA	Predefined Gemini voice, female pool	Female speakers	376	1128	1504
Leda	ar-MA	Predefined Gemini voice, female pool	Female speakers	399	1197	1596
Aoede	ar-MA	Predefined Gemini voice, female pool	Female speakers	381	1143	1524
Zephyr	ar-MA	Predefined Gemini voice, female pool	Female speakers	408	1224	1632

Noisy counts correspond to the three noisy variants generated from each clean file at 20 dB, 10 dB, and 5 dB SNR. Because voice usage is counted at the file level, totals across voices may exceed the number of released audio files when multiple voices appear in the same dialogue file.

The data/ folder contains four JSON files. dialogues_normal_clean.json and dialogues_scam_clean.json contain the clean text layer used for audio generation and alignment. Each scenario includes an id, a theme, an is_scam label, and a dialogue field containing the ordered turns. Each turn includes the speaker field s, emotion field e, Latin-script Darija text t, Arabic-script Darija text t_ar, and gender. dialogues_normal_public.json and dialogues_scam_public.json preserve the same scenario and turn structure, but sensitive spans in t and t_ar are replaced by structured placeholders. The public JSON files are intended for lower-risk inspection and redistribution; the clean JSON files are the text layer aligned with the released audio files.

The audio/ folder contains three main audio layers. audio/clean_normalized/ contains the clean normalized TTS-generated WAV files, organized by class into normal/ and scam/ subfolders. All clean audio files are mono, 24 kHz, PCM 16-bit WAV files. audio/noisy/ contains noisy WAV files generated from the clean audio using controlled environmental noise at three signal-to-noise ratio levels: 20 dB, 10 dB, and 5 dB. The noisy audio is organized by SNR level and class label. audio/noise_sources/ contains the 10 environmental noise files used for noise mixing, derived from the Moodist ambient sound library [13].

The metadata/ folder contains CSV metadata tables, taxonomies, manifests, and validation reports. scenarios_metadata_with_splits.csv provides one row per scenario, including dataset identifiers, class labels, attack-related metadata, risk-level fields, audio availability information, and train/validation/test split assignment. turns_metadata_emotion_normalized_with_splits.csv provides one row per dialogue turn and includes speaker, role, gender, emotion, normalized emotion class, text-level fields, scenario linkage, and split assignment. audio_manifest_normalized_with_splits.csv indexes the clean normalized WAV files and records file paths, labels, durations, sample rates, channel counts, format information, identifiers, checksums, and split assignment. noisy_audio_manifest_with_splits.csv indexes the noisy WAV files and records the clean source audio, noise source, SNR level, generated noisy path, audio properties, and split assignment.

The metadata/ folder also includes train_val_test_splits.csv, which provides the scenario-level split assignment; emotion_taxonomy.csv, which defines the normalized emotion categories; attack_taxonomy.csv, which defines the attack-type and social-engineering categories; noise_sources_metadata.csv, which documents the environmental noise files; data_dictionary.csv, which describes the released variables; repository_file_inventory.csv, which summarizes the main files in the repository; and validation reports documenting JSON schema validation, audio linkage, normalization, noisy-audio generation, split generation, checksum generation, and final release validation.

The scripts/ folder contains Python scripts used for reproducibility and release validation. validate_dataset.py validates the JSON dialogue structure against the schema. build_audio_manifest.py creates the clean audio manifest and verifies JSON–audio linkage. normalize_wav_format.py normalizes WAV files to mono, 24 kHz, PCM 16-bit format. add_noise_snr.py generates noisy variants at controlled SNR levels. normalize_emotions.py maps raw emotion labels to normalized emotion classes. build_metadata.py constructs scenario-level and turn-level metadata. make_splits.py creates the train/validation/test partitions and checks split leakage. compute_checksums.py computes SHA-256 checksums. final_release_validation.py performs final release-level validation.

The repository root contains README.md, dataset_card.md, ethics_statement.md, CITATION.cff, LICENSE, schema.json, checksums_sha256.txt, and release_notes_v1.0.md. README.md provides repository-level usage instructions. dataset_card.md summarizes dataset scope, structure, intended use, limitations, and ethical considerations. ethics_statement.md documents the synthetic nature of the data and the absence of real phone calls, real victims, or human-recorded speech. CITATION.cff provides citation metadata for the dataset. schema.json defines the expected JSON structure. checksums_sha256.txt provides SHA-256 checksums for release integrity verification. release_notes_v1.0.md documents the v1.0 release.

## 4 Experimental Design, Materials and Methods

**Fig. 2:**
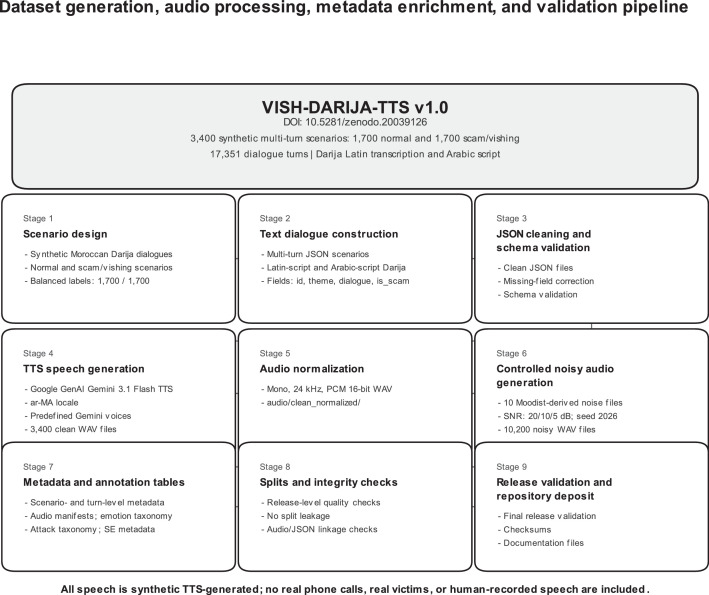
Dataset generation, audio processing, metadata enrichment, and validation pipeline for VISH-DARIJA-TTS v1.0. Synthetic Moroccan Darija normal and scam/vishing scenarios were structured as multi-turn JSON dialogues, cleaned and validated, converted into TTS-generated speech, normalized to WAV mono 24 kHz PCM 16-bit, augmented with controlled environmental noise at 20 dB, 10 dB, and 5 dB SNR, enriched with metadata and taxonomies, split into train/validation/test partitions, and validated with manifests, checksums, and release-level integrity checks.

The VISH-DARIJA-TTS v1.0 dataset was produced through a controlled synthetic data-generation pipeline covering scenario design, text dialogue construction, JSON cleaning and schema validation, TTS speech generation, audio normalization, controlled noisy-audio generation, metadata enrichment, split generation, checksum computation, and release validation (Fig. 2). The pipeline was designed to generate paired Moroccan Darija text and audio data without collecting real phone calls, victim recordings, or human-recorded speech.

The source dialogue text was synthetically produced and curated by the authors for this dataset. No real scam-call transcripts, customer-service logs, organizational records, social-media conversations, questionnaires, surveys, or secondary text datasets were used as source data. The original text layer consists of 3400 scenarios and 17,351 dialogue turns, corresponding to the released clean JSON layer before audio generation. Class labels were assigned by construction at the scenario level, with 1700 scenarios designed as normal conversations and 1700 scenarios designed as scam/vishing conversations.

The first stage consisted of designing synthetic multi-turn dialogue scenarios for two classes: normal and scam/vishing. The final release contains 3400 scenarios, with 1700 normal and 1700 scam/vishing scenarios. The normal class was constructed as non-fraudulent everyday dialogue scenarios, while the scam/vishing class was organized around voice-based social-engineering themes such as fake banking support, family impersonation, delivery scams, technical support scams, job-offer scams, legal or police impersonation, donation or inheritance scams, and other phone-fraud scenarios.

The second stage converted these scenarios into structured JSON dialogue files. Each scenario was assigned a numerical identifier, a theme, a dialogue object, and an is_scam label. Each dialogue turn was represented with a speaker field, an emotion field, Latin-script Darija text, Arabic-script Darija text, and a gender field used as role/voice metadata rather than as demographic information about a recorded participant. The text layer was represented in two scripts: Latin-script Darija and Arabic-script Darija, reflecting common written forms of Moroccan Darija in digital communication.

The clean JSON files, dialogues_normal_clean.json and dialogues_scam_clean.json, preserve the text used as the source layer for audio generation. The public JSON files, dialogues_normal_public.json and dialogues_scam_public.json, preserve the same scenario and turn structure but replace sensitive spans with structured placeholders. This public layer was generated to prevent redistribution of operational social-engineering templates while keeping scenario identifiers, turn order, labels, and metadata structure unchanged.

The JSON files were cleaned and validated before audio and metadata processing. The cleaning procedure preserved scenario identifiers and did not delete scenarios or reassign IDs. Structural corrections were applied only to fields that could be corrected safely, such as renamed fields or missing non-content metadata. Missing Arabic-script text fields were completed before final schema validation. The validated JSON structure requires each scenario to contain id, theme, dialogue, and is_scam fields, and each turn to contain s, e, t, t_ar, and gender fields. The script validate_dataset.py was used to validate the JSON dialogue structure against schema.json.

Synthetic speech was generated from the clean text layer using Google GenAI Gemini 3.1 Flash TTS [14]. This TTS model was selected because it enables scalable synthetic speech generation through predefined voices, supports a Moroccan Arabic locale configuration (ar-MA), and allows multi-speaker dialogue audio generation without recording human speakers. This choice was also consistent with the ethical design of the dataset, which avoids real victims, real phone calls, and human-recorded speech. The TTS input prompts were constructed from the clean JSON dialogue layer and preserved the scenario identifier, ordered dialogue turns, speaker labels, Darija text, and turn-level emotion/style information when available. The final voice pool included eight predefined Gemini voices configured with the Moroccan Arabic locale (ar-MA): Fenrir, Puck, Orus, Charon, Kore, Leda, Aoede, and Zephyr. Voice assignment followed a deterministic speaker-role and gender-based rule to introduce controlled speaker diversity across the synthetic multi-speaker dialogues. The generated audio was saved as WAV and then normalized to mono, 24 kHz, PCM 16-bit. Gemini TTS supports prompt-controllable expressive speech generation, including style, pace, tone, delivery, and emotional expression [14]. Accordingly, turn-level emotion/style information was used as prompt-level guidance when available. However, low-level provider-side parameters such as explicit pitch, speaking rate, and fine-grained prosody controls were not manually controlled. The voice-usage distribution is summarized in Table 3.

The clean audio files were normalized to a common technical format to ensure consistent reuse. The script normalize_wav_format.py converted the generated WAV files to mono, 24 kHz, PCM 16-bit format. Stereo sources were converted to mono, audio was resampled when needed, and the output files were written under audio/clean_normalized/ while preserving the class organization into normal/ and scam/ subfolders. The normalization process did not modify the clean JSON files or the released metadata tables.

The conversion to mono was applied to standardize all audio files into a single-channel format suitable for speech-processing and voice-fraud detection pipelines. This choice reduces channel-related variability, simplifies reproducible benchmarking, and ensures that all clean and noisy variants share the same technical format. The conversion was not intended to reproduce the full acoustic properties of a telephone network or telephony codec, but to provide a consistent and reusable audio representation across the released dataset.

Controlled noisy audio variants were generated from the clean normalized audio using add_noise_snr.py. The noise pool contained 10 environmental noise files derived from Moodist [13], covering airport, airplane, cafe, keyboard, library, office, paper, supermarket, train, and washing-machine conditions. For each clean audio file, three noisy variants were generated at target signal-to-noise ratio levels of 20 dB, 10 dB, and 5 dB. Noise assignment was deterministic, using a fixed seed of 2026 and a deterministic mapping based on the label, scenario identifier, and SNR level. The noise signal was aligned to the target clean signal length, scaled to match the requested SNR level, mixed with the clean signal, and written as mono, 24 kHz, PCM 16-bit WAV. Output amplitude was normalized after mixing to prevent clipping artifacts. The resulting noisy layer contains 10,200 WAV files under audio/noisy/.

The noisy variants model a single-channel global degradation condition in which the full dialogue is mixed with one environmental noise source at a controlled SNR level. The release does not simulate independent acoustic environments for each speaker or different background noise conditions at the two ends of a call. This design was chosen to keep the noisy benchmark deterministic, reproducible, and directly comparable across SNR levels.

Scenario-level and turn-level metadata were produced after the text and audio layers were validated. The script build_metadata.py generated scenario metadata and turn metadata from the clean JSON files and the audio manifests. For normal scenarios, social-engineering fields were set to baseline values such as none or low where applicable. For scam/vishing scenarios, fields such as attack_type, persuasion_strategy, requested_asset, impersonated_entity, victim_role, and risk_level were generated using rule-based or heuristic metadata extraction from themes and text fields. These metadata fields are intended as structured annotations for reuse and should be treated as heuristic labels for fine-grained analyses.

Emotion metadata were normalized using normalize_emotions.py. Raw emotion labels from dialogue turns were mapped to a controlled emotion taxonomy and written to turns_metadata_emotion_normalized_with_splits.csv. The original raw emotion information was preserved, while the normalized field provides a reduced set of controlled categories for downstream use. The metadata layer also includes attack_taxonomy.csv and emotion_taxonomy.csv to define the annotation categories used in the release.

Emotion labels were preserved as turn-level metadata and were used as prompt-level style and emotional-expression cues during TTS generation when available. This use is consistent with Gemini TTS documentation, which describes controllable speech generation through natural-language prompts for steering style, pace, tone, delivery, and emotional expression [14]. However, the dataset does not claim independent human perceptual validation of the generated emotional cues. Therefore, the emotion fields should be interpreted as structured dialogue-turn annotations and prompt-level synthesis cues, rather than as human-validated acoustic emotion labels.

Audio manifests were generated to link the text scenarios and audio files. build_audio_manifest.py indexed the clean normalized WAV files and verified correspondence between JSON scenario identifiers and clean audio files. The noisy audio manifest links each noisy file to its clean source audio, noise source, SNR level, label, split, and audio properties. These manifests provide the main linkage between scenario-level data, turn-level data, clean audio, noisy audio, and train/validation/test partitions.

Train, validation, and test splits were generated at the scenario level using make_splits.py. The final split distribution contains 2380 training scenarios, 510 validation scenarios, and 510 test scenarios. The split assignment preserves class balance: 1190 normal and 1190 scam/vishing scenarios in training, 255 normal and 255 scam/vishing scenarios in validation, and 255 normal and 255 scam/vishing scenarios in testing. All audio variants corresponding to the same scenario were kept in the same split to prevent leakage across partitions.

Release integrity was verified using checksum and validation scripts. compute_checksums.py generated SHA-256 checksums for release files, and final_release_validation.py checked the main release structure, metadata availability, audio readability, split consistency, checksum coverage, and absence of blocking release issues. The final repository includes checksums_sha256.txt, validation reports, data dictionaries, file inventories, metadata summaries, and release notes to support reproducible inspection and reuse.

Data validation and quality-control checks. The release was validated using automated data-integrity and technical quality-control checks rather than downstream model-performance evaluation. The validation pipeline checked JSON schema compliance, scenario identifier consistency, class-label consistency, audio readability, audio duration constraints, sample rate, channel count, PCM format, clean-to-noisy linkage, train/validation/test leakage, checksum coverage, and absence of blocking release issues. These checks were designed to verify the internal consistency, technical usability, and reproducibility of the released dataset. Table 4.

**Table 4:** Release validation and quality-control checks.

Check category	Validation target	Result
JSON schema	Required scenario and turn fields in clean and public JSON files	Passed
Scenario identifier consistency	Consistent scenario identifiers across JSON files, metadata tables, and audio manifests	Passed
Class-label consistency	Scenario-level normal and scam/vishing labels	Passed
Audio readability	Clean and noisy WAV files readable by the validation scripts	Passed
Audio format	Mono, 24 kHz, PCM 16-bit WAV format	Passed
Audio duration constraints	Valid duration values recorded for released clean and noisy audio files	Passed
Clean-to-noisy linkage	Noisy files linked to their corresponding clean source files and noise sources	Passed
Split leakage control	All clean and noisy variants of the same scenario kept in the same train/validation/test split	Passed
Checksum coverage	SHA-256 checksums generated for released files	Passed
Final release validation	No blocking release issues after final validation	Passed

## Limitations

The dataset has limitations related to its controlled synthetic design and intended scope of use. First, the audio files are generated using text-to-speech synthesis and do not contain real telephone-channel recordings, spontaneous caller behaviour, real victims, or human-recorded speech. Therefore, the acoustic and interactional variability of real-world scam calls is not fully represented. Although Gemini TTS supports prompt-controllable expressive speech generation, including style, pace, tone, delivery, and emotional expression [14], the generated audio should not be interpreted as an independently human-validated reproduction of emotional or persuasive cues observed in real social-engineering calls. The turn-level emotion labels were used as prompt-level synthesis cues when available, but no formal perceptual study was conducted to verify that listeners consistently perceive the intended emotions in the generated speech. Therefore, the audio layer is best suited for controlled benchmarking and reproducible method development rather than for claims about the full acoustic realism of real-world fraudulent calls. Second, the dataset contains Darija in Latin and Arabic scripts, but it does not provide token-level annotation for code-switching, Modern Standard Arabic, French borrowings, or dialectal variants. Finally, the noisy audio layer is based on 10 environmental noise files and three fixed SNR levels, which provide controlled acoustic conditions but do not cover all real telephone or environmental noise situations. In addition, the noisy layer represents a single-channel global noise condition and does not model separate acoustic environments or different noise intensities for each speaker endpoint.

## Ethics Statement

The authors have read and followed the ethical requirements for publication in Data in Brief. The current work does not involve human subjects, animal experiments, or data collected from social media platforms.

The VISH-DARIJA-TTS v1.0 dataset does not contain real phone calls, real victims, human-recorded speech, recordings of identifiable individuals, or personal data collected from participants. All dialogue scenarios were synthetically constructed as normal or scam/vishing conversations, and all speech files were generated using text-to-speech synthesis. The gender field included in the dataset is used only as role/voice metadata for synthetic audio generation and does not represent demographic information about human participants.

Because no human subjects were recruited, no human speakers were recorded, and no real victim conversations were collected, informed consent for audio recording was not applicable to this dataset. Ethical committee approval was not applicable to the released dataset because it contains no human-subject recordings, no identifiable participant data, and no personal data collected from individuals. The dataset was designed for defensive cybersecurity research, teaching, benchmarking, and reproducible experimentation on vishing and voice-based social engineering.

To reduce misuse risk, the public JSON layer replaces sensitive spans such as payment amounts, transfer services, names, social-media accounts, locations, OTP codes, bank-card references, and identity-document references with structured placeholders. The dataset should not be used to conduct fraud, train operational scam scripts, impersonate real organizations or individuals, or deploy systems that facilitate social engineering.

## CRediT Author Statement

**Yasser Hmimou:** Conceptualization, Methodology, Data curation, Formal analysis, Investigation, Writing – original draft, Writing – review & editing, Project administration. **Nada Jabel:** Software, Data curation, Validation, Investigation, Writing – review & editing. **Soufiane Ameur:** Writing – review & editing, Validation.**Mohamed Tabaa:** Supervision, Writing – review & editing.

## Data Availability

zenodoVISH-DARIJA-TTS: A Synthetic Moroccan Darija Text-Audio Dataset for Vishing and Voice-Based Social Engineering Detection (Original data)
